# Editorial: Endophenotypes for Schizophrenia and Mood Disorders: Implications from Genetic, Biochemical, Cognitive, Behavioral, and Neuroimaging Studies

**DOI:** 10.3389/fpsyt.2016.00083

**Published:** 2016-05-11

**Authors:** Błażej Misiak, Dorota Frydecka, Janusz K. Rybakowski

**Affiliations:** ^1^Department of Psychiatry, Wroclaw Medical University, Wroclaw, Poland; ^2^Department of Genetics, Wroclaw Medical University, Wroclaw, Poland; ^3^Department of Adult Psychiatry, Poznan University of Medical Sciences, Poznan, Poland

**Keywords:** endophenotype, schizophrenia, mood disorders, psychosis, genetics

**The Editorial on the Research Topic**

**Endophenotypes for schizophrenia and mood disorders: implications from genetic, biochemical, cognitive, behavioral, and neuroimaging studies**

Schizophrenia and mood disorders represent complex phenotypes that are characterized by multidimensional psychopathology and biological underpinnings. After many years of clinical observations, it has been recognized that there are several intermediate phenotypes between schizophrenia and mood disorders that share common clinical characteristics. This observation has provided grounds for the concept of endophenotypes, crossing traditional diagnostic boundaries and opening perspectives for better understanding of mental disorders. Indeed, the term endophenotype refers to a measurable construct that bridges a gap between phenotype expression and genetic variability. This intermediate phenotype construct must meet four characteristics: the association with illness in the population, heritability, state-independent manifestation (expression of the phenotype regardless of the illness activity), and cosegregation with illness in families (1). Consequently, a number of promising genetic, biochemical, cognitive, behavioral, and neuroimaging endophenotypes emerged providing a broader insight into the nature of schizophrenia and mood disorders.

In this research topic, a few promising candidates of clinical, biochemical, and neuroimaging endophenotypes have been proposed. Grant describes various aspects of schizotypy and proposes that schizotypy itself might be a suitable endophenotype of schizophrenia. Following the definition of endophenotype developed by Gottesman and Gould (1), the author indicates that schizophrenia patients have high levels of schizotypy that are even higher than in other psychotic disorders. Grant also provides evidence that schizotypy is highly heritable and cosegregates with schizophrenia with the illness in families. Additionally, it has been reported that healthy relatives of schizophrenia patients have higher levels of schizotypy than healthy individuals with negative family history of schizophrenia. Finally, Grant shows that schizotypy is a stable trait with high test–retest reliability in patients with schizophrenia. However, it should be noted that higher levels of schizotypy have been also reported in patients with bipolar disorder (BD) (2–4). Therefore, future studies should focus on determining the validity of schizotypy together with its specific dimensions as an endophenotype of schizophrenia or BD.

In two studies, the role of structural and functional brain alterations as potential endophenotypes of psychosis dimension has been investigated. In the large and multisite bipolar–schizophrenia network on intermediate phenotypes (B-SNIP) study, Wang et al. found that functional alterations in a prefrontal–striatal–thalamic–cerebellar network and structural disturbances in the default mode network are common abnormalities across various psychotic disorders. Based on the data from B-SNIP study, Soh et al. performed joint independent component analysis of awake EEG frequency activity and MRI gray matter volumes in patients with psychotic disorders. Authors found a single component differentiating schizophrenia patients and healthy individuals. Specifically, they reported increased posterior alpha activity associated with decreased volume in inferior parietal lobe, supramarginal, parahippocampal gyrus, middle frontal, inferior temporal gyri, and increased volume of uncus and culmen in patients with schizophrenia.

In the last article, Kim et al. in their mini review proposed that phospholipase C-β1 (PLC-β1) hypofunction might be an endophenotype of schizophrenia. This enzyme is involved in phosphoinositide signaling, which is one of the main G-protein-related pathways in the central nervous system. It has been reported that PLC-β1^−/−^ mice express several negative-like symptoms and cognitive deficits that resemble those observed in schizophrenia (5–7). In addition, lower PLC-β1 has been found in several brain regions of patients with schizophrenia (8). Interestingly, the role of aberrant phospholipase C-γ1 (PLC-γ1) signaling has been also implicated in the pathophysiology of BD [for review, see Ref. (9)). In addition, polymorphism of the PLC-γ1 gene has been found to predict response to lithium treatment (10). A recent analysis of data from the genome-wide association study (GWAS) also revealed that genetic variation in phospholipase C signaling might be associated with a risk of BD (11). Therefore, more research is needed to disentangle the role of impaired phospholipase C signaling as an endophenotype for both schizophrenia and BD.

Although a number of candidate endophenotypes for schizophrenia and psychotic disorders have been proposed in this research topic, studies replicating these findings in large samples are required. In addition, future studies must look at these findings in a broader context of endophenotype criteria and verify how well they suit their original purpose – linking less complex disease phenotype to its genetic underpinnings. It is important to note that studies investigating endophenotypes should take into account that psychosis dimension is itself a heterogeneous construct. Indeed, psychotic symptoms associated with schizophrenia have different features compared to those observed in BD. For instance, most psychotic features associated with manic episodes tend to have a different, usually self-limited and self-remitting course. These symptoms appear in most severe episodes of BD. On the contrary, psychotic symptoms in schizophrenia have a more long-lasting course. This observation give rise to the conclusion that more objective and reliable endophenotypes offer a prospect of defining more etiologically homogenous subgroups of human psychopathology and thus facilitate our understanding of the basis and causal pathways of mental disorders. Finally, the existence of relevant endophenotypes should initiate discussion about reappraisal of the research diagnostic criteria beyond traditional diagnostic systems to enable better understanding of mechanisms underlying schizophrenia and mood disorders.

## Author Contributions

All author listed have made substantial, direct, and intellectual contribution to the work and approved it for publication.

## Conflict of Interest Statement

The authors declare that the research was conducted in the absence of any commercial or financial relationships that could be construed as a potential conflict of interest.
